# “Big Food,” the Consumer Food Environment, Health, and the Policy Response in South Africa

**DOI:** 10.1371/journal.pmed.1001253

**Published:** 2012-07-03

**Authors:** Ehimario U. Igumbor, David Sanders, Thandi R. Puoane, Lungiswa Tsolekile, Cassandra Schwarz, Christopher Purdy, Rina Swart, Solange Durão, Corinna Hawkes

**Affiliations:** 1 School of Public Health, University of the Western Cape, Bellville, South Africa; 2 Bloomberg School of Public Health, Johns Hopkins University, Baltimore, Maryland, United States of America; 3 Department of Dietetics, University of the Western Cape, Bellville, South Africa; 4 Centre for Food Policy, City University, London, United Kingdom

## Abstract

In an article that forms part of the *PLoS Medicine* series on Big Food, Corinna Hawkes and colleagues provide a perspective from South Africa on the rise of multinational and domestic food companies, and argue that government should act urgently through education about the health risks of unhealthy diets, regulation of Big Food, and support for healthy foods.

**Summary Points**

- In South Africa, as in other jurisdictions, “Big Food” (large commercial entities that dominate the food and beverage environment) is becoming more widespread and is implicated in unhealthy eating.
- “Small food” remains significant in the food environment in South Africa, and it is both linked with, and threatened by, Big Food.
- Big Food in South Africa involves South African companies, some of which have invested in other (mainly, but not only, African) nations, as well as companies headquartered in North America and Europe.
- These companies have developed strategies to increase the availability, affordability, and acceptability of their foods in South Africa; they have also developed a range of “health and wellness” initiatives. Whether these initiatives have had a net positive or net negative impact is not clear.
- The South African government should act urgently to mitigate the adverse health effects in the food environment in South Africa through education about the health risks of unhealthy diets, regulation of Big Food, and support for healthy foods.

*This article was commissioned for the* PLoS Medicine *series on Big Food that examines the activities and influence of the food and beverage industry in the health arena.*

## Introduction

Despite continuing high levels of underweight and nutritional deficiencies [1], overweight and obesity among both adults and children is a rapidly growing public health problem in South Africa [2],[3],[4],[5]. In 2000, an estimated 36,504 deaths (7% of all deaths) in South Africa were attributed to excess body weight [6], and in 2004 non-communicable diseases (NCDs) linked to dietary intake—cardiovascular diseases, diabetes mellitus, cancers—together with respiratory diseases contributed 12% of the overall disease burden [7].

Paralleling this increase in overweight/obesity, there has been a steady increase in the per capita food supply of fat, protein, and total calories in South Africa [8],[9] and salt intake appears to also be in excess of recommended levels [10]. These changes of nutrient intake appear to be associated with changes in dietary patterns. So, for example, a study of adults in the North West Province showed a shift with increasing wealth from a traditional high carbohydrate–low fat diet (in which maize made the largest contribution to energy intake) to a higher-fat diet in which maize was replaced by red meat and other cereal foods [11].

In recent years, there has also been an increase in the sales of almost all categories of packaged foods in South Africa (Table 1). For example, sales of snack bars, ready meals, and noodles all rose by more than 40% between 2005 and 2010. In addition, a recent assessment of the consumption of street food (sold by vendors) and fast food (from formal fast food outlets) revealed that, nationally, 11.3% of the population bought food from street vendors and 6.8% bought food from fast food outlets at least two times a week [12]. South Africans are also increasing their consumption of soft drinks. Compared with a worldwide average of 89 Coca-Cola products per person per year, in 2010 South Africans consumed 254 Coca-Cola products per person per year, an increase from around 130 in 1992 and 175 in 1997 [13],[14]. In 2010, up to half of young people were reported to consume fast foods, cakes and biscuits, cold drinks, and sweets at least four days a week [4]. Carbonated drinks are now the third most commonly consumed food/drink item among very young urban South African children (aged 12–24 months)—less than maize meal and brewed tea, but more than milk [15].

**Table 1:** Volume of sales of select leading categories of packaged foods, 2010, and rate of increase 2005–10.

Category of Packaged Foods	Subcategory	Sales Volume*	Rate of Change of Sales Volume (%), 2005–10
Bakery		2009.3	16.2
Meal solutions		547.2	18.5
	Canned/preserved food	241.8	16.2
	Frozen processed food	102.1	18.2
	Chilled processed food	95.9	−2.8
	Sauces dressings and condiments	88.1	27.0
	Ready meals	70.1	43.1
	Soup	11.1	32.6
Impulse and indulgence products			
	Confectionery	119.4	16.3
	Sweet and savoury snacks	87.9	27.5
	Snack bars	1.9	42.6
	Ice cream	76.0	14.7
Dried processed food		345.4	−2.8
Pasta		62.9	35.0
Noodles		7.4	44.5
Oils and fats		343.6	14.9
Meal replacement		0.6	9.6
Spreads		28.8	23.9

Source: Euromonitor 2011 [17].

*: in thousand tonnes, except for ice cream, which is million litres.

**Figure 1:**
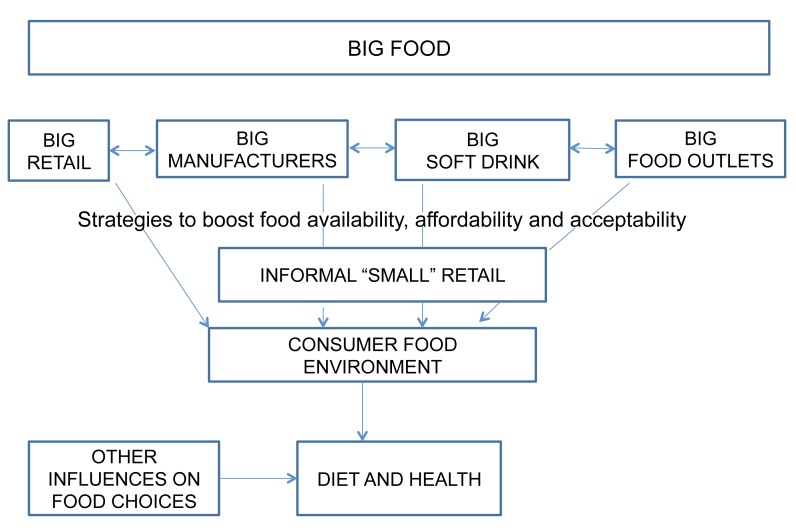
Hypothesized link between Big Food and the consumer food environment.

It can be hypothesised that various strategies adopted by “Big Food” to increase the availability, affordability, and acceptability of their products have contributed to these dietary changes in South Africa and to the increased burden of obesity and NCDs (Figure 1). In this context, in this article we provide an overview of “Big Food” in South Africa. We use the term “Big Food” as shorthand for large commercial entities—both multinational and national—that increasingly dominate key components of the food and beverage environment. We include companies that have an identity with consumers—manufacturers, retailers, and food outlets—rather than agribusiness and primary processors. Although many authors have written on Big Food in the US, the UK, and other developed nations, much less has been written about their operations and practices in developing countries experiencing significant transitions. As such, this article contributes to filling a gap in the literature and provides similar nations with a process for examining the role of Big Food in health and nutrition. Our article draws on information published in the academic literature, reviews of food industry documents, data compiled by market research agencies, and data from pilot studies conducted by researchers at the University of the Western Cape, South Africa.

We first present data on the presence of Big Food in South Africa, then examine some of the strategies used by large food corporations to change the consumer food environment in South Africa. We argue that these strategies aim to alter the availability, affordability, and acceptability of foods produced and sold by Big Food (Figure 1). Finally, we discuss the responses to health concerns made by both Big Food and the South African government, and briefly explore the policy implications.

## Big Food in South Africa

### Packaged Foods

Although there are over 1,800 food manufacturing companies in South Africa, Big Food manufacturers account for a disproportionately large amount of sales [16]. The largest ten packaged food companies in South Africa account for 51.8% of total packaged food sales (Table 2), whereas artisanal packaged processed foods (products sold at the site of production, commonly bakery products), contribute only 7.3% of total sales [17],[18]. This is greater than the global average (globally in 2007, ten companies accounted for around 26% of the processed foods market) [19].

**Table 2:** Packaged Food Company Shares in South Africa, 2009.

Rank	Company	Location of Company Headquarters	Contribution to Total Packaged Food sales (%)	Examples of Product Types
1	Tiger Brands Ltd	South Africa	17.2	Milling and baking, groceries, confectionery, beverages, value added meat products, fruit and vegetables, products for the food services sector
2	Unilever Group	UK/Netherlands	4.9	Spices, sauces, dressings, margarine, teas, syrup and food solutions
3	Parmalat Group	Italy	4.8	Dairy products including milk, yoghurt, ice cream and cheese, fruit juices
4	Nestle SA	Switzerland	4.6	Baby foods, drinks, breakfast cereals, chocolate, confectionery, coffee, dairy products, ice cream
5	Clover Ltd	South Africa	4.6	Dairy products, desserts, beverages such as fruit juices, nectars and ice teas
6	Dairybelle (Pty) Ltd	South Africa	4	Dairy products, fruit juices
7	Pioneer Food Group Ltd	South Africa	3.7	Baking aids, tea/coffee, breakfast cereals, biscuits, condiments, juices and acidic drinks, dried fruits, eggs
8	Cadbury Plc (bought by Kraft in 2011)	UK/US	2.8	Chocolate, candy, gum, biscuits, coffee, other grocery
9	AVI Ltd	South Africa	2.8	Coffee, tea, biscuits, potato chips, frozen fish and seafood products
10	PepsiCo Inc	US	2.4	Drinks, savoury snacks

Source: Derived from Euromonitor*, 2011 [17] In: Alexander et al 2011 [18]; company websites.

*: Euromonitor does not collect data on the informal sector (defined as sales that are not taxed).

Five of the top ten food manufacturing companies are South African, three of which have an international presence. Pioneer Foods, for example, has a presence in four south and east African nations, is a leading exporter, and has joint ventures with Heinz (US) (as Heinz Foods South Africa) and with a UK-based ingredients company. Many other South African companies have likewise developed joint ventures with foreign companies, such as Simba with Frito-Lay (PepsiCo, USA) and Clover with Danone Groupe (France).

### Soft Drinks

The top ten soft drink companies account for 79% of the total soft drink sales in South Africa. Three companies – Coca-Cola Co., PepsiCo Inc., and Danone Groupe – account for 64.7% of the market between them, with the other top companies each contributing less than 3.5% [20]. All three market leaders are major transnational companies but are linked with South African companies. For example, Coca-Cola is bottled by SABMiller, a division of AVI, and PepsiCo is manufactured by Pioneer Foods under license.

By far the largest player is Coca-Cola (49.8% of total soft drink sales). Coca-Cola has been a dominant presence in South Africa for decades, including during the apartheid era when it distributed its products from Swaziland. After apartheid ended, Coca-Cola became a leading investor in the country [13].

### Food Service Outlets

In 2010, there were 8,661 fast food outlets in South Africa, 4,991 of which were owned by fast food chains, the remainder being independent outlets (Table S1). [17]. Although there are considerably more street stalls/kiosks than fast food outlets in South Africa, the number of transactions from fast food chains has increased significantly over past decades [13]. The fast food chains are dominated by South African companies that pre-date the presence of foreign transnationals. For example, Nando's (chicken) was established years before Yum! (which owns KFC) entered the market in 1994. Today, eight of the largest ten food service companies are South African. Two of these have expanded internationally: Famous Brands purchased Wimpy UK in 2007, and Nando's has worldwide franchising operations.

Many of these fast food chains are linked with large soft drink companies through agreements concerning the drinks served in the restaurants: Wimpy and Nando's, for example, serve Coca-Cola products exclusively.

### Retailers

One of the most dramatic changes in Big Food in South Africa has been the rise in supermarket retailers over past decades. Chain supermarkets now control over half of the retail share of the food market [20], which is dominated by four major chains (Shoprite, Pick n Pay, Spar, and Woolworths), all of which are South African (Table S2). Each of these companies owns several different supermarket brands, and all have expanded into other African countries.

### Imports

South Africa is a net importer of various agricultural products and foods. In the past fifteen years, there has been a marked rise in imports of processed products. For example, the value of imported “Bread, Pastry, Cakes, Biscuits and Other Baker's Wares” increased from approximately R5 million (US$ 714,000; all conversions are based on the exchange rate at the relevant time) in 1992 to almost R250 million (US$ 36 million) in 2006 [21]. In addition, the import of ingredients used in processed foods has increased. For example, imports of whey, a by-product of cheese production that is used in baked products and sweet snacks, increased from R15 million (US$ 2 million) in 1993 to R80 million (US$ 11 million) in 2007 [13].

## Big Food Strategies to Alter the Consumer Food Environment

Big Food manufacturers have worked to increase the market share for their products in South Africa and to increase per capita consumption by making their foods more available, affordable, and acceptable (Figure 1).

### Making Their Foods More Available

Rising sales of packaged foods from large food manufacturers have gone hand-in-hand with the increase in market share held by Big Food retailers. Supermarket outlets have displaced traditional food retailers such as small convenience stores, public markets, and “spazas” (small informal shops often run from homes) as the primary place from which South Africans purchase their food in both urban and rural areas [22]. For example, a case study in the rural Ciskei region of the Eastern Cape found that 64.8% of households in villages used supermarkets [22]. The expansion of supermarkets into rural (and other lower income) areas has been greatly facilitated by their retail management and procurement models, which have allowed them to out-compete local wholesalers and small retailers on cost and quality in virtually all product offerings [23].

Big Food manufacturers depend on formal retail chains to make their manufactured products available [16]. As well as being the main source of staple products, supermarkets are the main sales channel for “non-essential” packaged foods such as “meal solution” products made by large food corporations. However, strategies to increase the availability of products made by large food manufacturers have also involved the informal retail sector. Informal traders sell soft drinks, dairy products, bakery products, and snacks such as chips (crisps) in urban settlements and rural areas [24], and Coca-Cola, in particular, has worked hard to increase product sales and consumption through this channel. Coca-Cola developed a strategy in the 1990s to “double soft drinks sales” by “building up per capita consumption” [25]. Making their products more available through informal stores was a crucial part of the strategy. They developed incentives for people to set up informal outlets in the townships, such as providing trolleys, lighting boards, point-of-sale display materials, and refrigeration equipment, and delivered the products direct to the stores [17]. By 2005, around 95% of spazas were selling Coca-Cola products, with the drinks forming a large proportion of the turnover of these small outlets [26].

Similarly, although the amount of street foods purchased remains almost twice the amount purchased from formal fast food outlets [12], fast food chains have developed aggressive expansion strategies to make their products more available. For example, McDonald's only entered the South African market in 1995 but by 2001, it had 103 outlets. It now has 161 [13]. According to the company “South Africa is one of the most successful markets in McDonald's international history. A record was set when South Africa opened 30 restaurants in just 23 months, at one stage opening 10 restaurants in 78 days” [27].

### Making Their Foods More Affordable

The presence of supermarket chains has also had an impact on food prices. When purchased through informal retailers, who usually procure their goods from wholesalers, the mark-up on some processed food products is as high as 39% [22]. This mark-up is frequently attributed to transport and distribution costs and to maintaining profit margins, but may also reflect the monopoly position of local stores in poor communities [22]. Big Food retailers have developed a completely different procurement strategy based on cutting out the traditional wholesalers, consolidating their suppliers, and dealing with larger volumes [23]. Today, as a result, food prices are lower in supermarkets than in traditional retail outlets, which makes both staple foods and the packaged foods produced by large manufacturers more affordable to local populations.

Notably, however, healthier foods, which are more readily available in supermarkets than in small shops, typically cost between 10% and 60% more in supermarkets than less healthy foods when compared on a weight basis, and between 30% and 110% more when compared on the cost of food energy [28]. Refined cereals and foods with added sugar and fat are among the lowest-cost sources of energy in rural supermarkets, thus making nutrient-poor products such as biscuits, margarine, and oil-heavy snacks an effective means to cheaply consume energy while adding new and varied tastes to rural diets. Nutrient-dense foods such as lean meat, fish, fruit, and vegetables generally cost far more than these inexpensive processed food products.

The price differentials among foods manufactured in South Africa by large food corporations, small domestic companies, and imported equivalents have not been comprehensively studied. However, a small study of imported highly processed products (many of them produced by Big Food outside of South Africa) and locally produced equivalents (many of them made by South Africa's largest food company, Tiger Brands) in the Shoprite, Pick n Pay, and Spar supermarket chains suggests a complex picture [29]. For all categories of processed food except breakfast cereals, the imported products were cheaper than the local equivalents in terms of average cost per 100 g, but the price differentials varied widely. So, for instant meals, the average cost per 100 g of imported products was R0.62 (US$ 0.09) less than locally produced products, whereas the price differential for salty snacks was R1.49 (US$ 0.21). Interestingly, although the imported products appear to be cheaper, their nutritional content appears to be better; products produced by domestic food companies tend to have higher sodium and total and saturated fat content than the imported equivalents (unpublished data) [29].

### Making Their Foods More Acceptable

All the components of Big Food have marketing strategies to make their foods more acceptable to the South African population. Food manufacturers work in conjunction with supermarkets to develop sales promotions for their products. PepsiCo, for example, has specific promotions on its website for each of the large retailers [30]. Competitions are also very common on manufacturer's websites. For example, the websites of both Nestlé South Africa and Cadbury South Africa (now owned by Kraft Foods) contain competitions for their main chocolate brands [31],[32].

Packaging is also used to promote products, with one of the more recent trends being statements on packages designed to appeal to health-conscious consumers. For example, Albany bread (a Tiger Brands company) promotes its products on the basis of health with statements like: “It's a great source of fibre, and fortified with vitamins and minerals, and it's cholesterol free” and some of its products carry a “low GI” symbol [33]. Similarly, Nestlé claim “Simple Goodness High in Fibre” on Maggi 2-minute noodle packets [34], and Rama margarine (a Unilever brand) is sold with the claim “Rama is a vital energy source, highly fortified with 8 GoodStart™ Vitamins, making it a highly nutritious margarine and spread” [35].

Television advertising of food is widespread. According to data from the South African Advertising Research Foundation, between 2003 and 2005, children 7–15 years old watched 2.5 hours of TV per day, and were exposed to 24 minutes of advertising per day [36]. A study undertaken in 2006 that recorded 37.5 hours of children's TV programming reported that 16% of the advertisements during this programming were for food products, and that 55% of these food-related advertisements were for foods of poor nutritional value such as refined breakfast cereals, sweets, and high-sugar drinks [37]. In a recent pilot survey, researchers at the University of Western Cape identified eight food advertisements in 7 hours of children's TV programming on the national TV channel SABC1 that they recorded in January 2012—four for sweets, one for refined breakfast cereal, two for tea, and one for a milk product (unpublished data).

Finally, although all Big Food engages in marketing in South Africa, expenditure on marketing campaigns tends to be higher by the transnationals than by South African companies. McDonald's and KFC, for example, spent more than four times than Nando's advertising their products in 2001 [13].

## Response to Health Concerns

Although it is not clear to what extent Big Food can be implicated in the changing diets and changing rates of obesity and related diseases in South Africa, it is perceived as at least partially culpable because of the strategies described in the previous section. Indeed, the South African Minister of Health, Dr. Aaron Motsoaledi, recently stated that “…Africans are eating more and more junk processed foods instead of their traditional diet,” and wants to regulate junk food starting with reducing salt in bread and eliminating trans fats [38].

In response, Big Food has developed corporate social responsibility (CSR) programs that involve health. All the major food manufacturers and retailers in South Africa have active CSR programs, with Shoprite, Pick n Pay, Spar, and Woolworths having the most active examples. The focus of these programs varies, but they often have a strong focus on nutrition education. Fast food chains tend to focus more on sponsoring local sports teams and sports tournaments. For example, McDonald's was an official sponsor during the FIFA World Cup, which prompted critical comments by health and consumer organizations [39].

The generally weak national response to the influential promotion of these products is illustrative of the rudimentary status of South Africa health policy regarding regulation of the food environment. However, Dr. Motsoaledi recently convened a National Consultation on NCDs. One aspect of this consultation was a recognition of important “upstream” factors, including the changing food environment [40]. The government has also developed a number of limited policy responses in the key areas of product labelling, marketing to children, and product reformulation. Big Food has also responded in these key policy areas [41].

### Product Labelling

Amidst concerns that claims made on food are “superlatives and often ludicrous” [42], the South African government changed the regulations on food product labelling in 2010. The Regulations Relating to the Labelling and Advertising of Foodstuffs, No. R. 146 of the Foodstuffs, Cosmetics and Disinfectants Act, 1972 came into effect on March 1, 2012 [41],[43]. Under the regulations, nutritional labelling remains voluntary unless a claim is made on the product. However, the regulations require a standard format for the nutritional label when used, and statements such as “A source of,” “High in,” “Low in,” “Virtually free of,” or “Free of” specific nutrients are only permitted if strict criteria are met. Manufacturers may no longer use descriptive words such as x% fat-free, nutritious, healthy, healthful, wholesome, complete nutrition, or balanced nutrition.

On a voluntary basis, several Big Food companies, including Tiger Brands, Spar, and Coca-Cola, use Guideline Daily Amount (GDA) labelling. These labels, like those used in other developed and developing countries, detail the quantity of specific nutrients in the products and the recommended daily allowance. Many Big Food companies also provide nutritional information on their websites.

### Food Marketing to Children

In 2007, the South African government included restrictions on food advertising to children under 16 years old in a draft Foodstuffs, Cosmetics & Disinfectants Act. The regulations envisaged that certain foods, categorized as “non-essential to a healthy lifestyle” (e.g., carbonated drinks, confectionery, potato chips [crisps], certain fast foods), would be prohibited from being advertised or promoted to children in any manner. The regulations would also have prohibited cartoon-type characters, puppets, animation, tokens, or gifts in the advertisement or promotion of any foodstuff to children, and some foodstuffs such as soya and dairy products would have had to carry labels advising consumers to use the product in moderation and warning that excessive consumption on a regular basis may lead to poor health.

However, the Department of Health put the implementation of its proposed regulations (which were reported to have caused “much heated debate in view of the many severe restrictions that they contained and their potential far-reaching effects” [44]) on hold, deciding instead to wait for the publication of the 2010 WHO Set of Recommendations on Marketing Food and Non-Alcoholic Beverages to Children [45]. The South African government has yet to publish any regulations of its own, although it is reported that it is still deliberating on its intended course of action [46].

Meanwhile, Big Food has taken voluntary action on food marketing to children. In 2009, the South Africa Pledge on Marketing to Children was established. An initiative of the Consumer Goods Council of South Africa, it has 24 signatories, including food manufacturers, retailers, and fast food chains. The Pledge is similar to pledges made in Europe (EU Pledge) and elsewhere, but covers only advertising on TV and in schools to children under age 12 [47]. Companies do not appear to have made specific commitments to the pledge, and no monitoring report has been released.

### Product Reformulation

The Ministry of Health is currently developing a salt reduction initiative that will gradually reduce salt levels in several highly consumed products, including bread, gravies and spices, brine chicken, cereal, margarine, and salty snacks, over a ten-year period [48]. It has been estimated that reducing the salt content in bread alone could prevent 6,500 deaths [48].

The Department of Health also limited the use of artificial trans fats to a maximum of 2% of oil or fat in all foods in its Regulations Relating to Trans Fats in Foodstuffs No, R 127 published in 2011[49]. These regulations specifically refer to synthetic trans fats and apply to all foodstuffs sold, manufactured in or imported into South Africa, as well as food prepared in restaurants, fast food outlets, and the catering industry. Woolworths were reported to be the first retailer to have removed trans fats from their entire brand product range (in 2007) [50], and KFC South Africa says it eliminated all trans fatty acids from its food products in August 2009 [51].

Big Food is also taking voluntary action. Unilever South Africa, for example, states that: “We are committed to improving the fat composition of our products by reducing saturated fat as much as possible and increasing levels of essential fats. All of our Flora margarine and our Rama Original and Spread for Bread tubs already contain less than 33% saturated fat as a proportion of total fat” [47]. Fast food outlets in South Africa also have salads and other “healthier” items on their menus.

## Conclusions

The combined processes of rapid urbanisation, concentration of ownership of food production and distribution, and globalisation of food trade have resulted in rapid changes in the South African food environment. This article has focused on Big Food and attempted to bring together what is known about large food corporations in South Africa in the context of concerns about unhealthy eating and associated ill health. Although it provides an incomplete picture, it yields the following observations about Big Food in South Africa:

- It has strong similarities with Big Food in other jurisdictions: it's big, it's getting more widespread, and it's implicated in unhealthy eating.
- It consists of large packaged food and soft drink manufacturers, large retailers, and food outlets. These different components of Big Food are linked through various pathways. While “small food” remains significant in the food environment in South Africa, it is both linked with, and threatened by, Big Food.
- It involves both “foreign” transnationals and South African companies. Many of the South African Big Food companies, particularly the supermarket chains, have invested in other African nations and around the world. This suggests that South African Big Food may become a more important global player in years to come and that global attention on Big Food should focus both on companies whose headquarters are in North America and Europe and on companies whose headquarters are in developing nations.
- Supermarkets must not be forgotten as a key component of Big Food in South Africa, where they constitute a major sales channel for the products produced by food manufacturers.
- Big Food in South Africa is increasingly developing “health and wellness” initiatives. The outcome of these initiatives is not yet clear: they may have a net positive impact but if they offset more rigorous government action they may have a net negative impact.

It is clear that urgent action is required to mitigate the adverse health effects of the changing food environment in South Africa. We suggest that this action should include a combination of accelerated efforts to educate the public about the adverse consequences of consuming easily available but unhealthy foods and greater regulation of Big Food and the strategies it employs to increase the availability, affordability, and acceptability of foods associated with unhealthy diets. The policy response to Big Food should also recognise the role of local and possibly subcontinental governance (e.g., the Southern Africa Development Community governments) in addressing the issue.

In conclusion, we suggest that the South African government should develop a plan to make healthy foods such as fruit, vegetables, and whole grain cereals more available, affordable, and acceptable, and non-essential, high-calorie, nutrient-poor products, including soft drinks, some packaged foods and snacks, less available, more costly, and less appealing to the South African population. Some of these approaches may require engagement with Big Food. But elsewhere, clear rules and regulations are needed. Discussions about the regulation of promotional activities and about imposing taxes on unhealthy food products would be a good place to start.

## Supporting Information

Table S1 Ten largest food service companies in South Africa.
(DOC)

Table S2 Supermarket value sales, number of outlets, and company shares by value in South Africa 2007–2009.
(DOC)

## Abbreviations

CSR: corporate social responsibility
NCD: non-communicable disease
