# Supplementary material for: “Big Food,” the Consumer Food Environment, Health, and the Policy Response in South Africa
Source: PLoS Med. 2012 Jul 3;9(7):e1001253. doi: 10.1371/journal.pmed.1001253 (PMC3389030; doi:10.1371/journal.pmed.1001253)
Supplement: Table S1 — Ten largest food service companies in South Africa. (DOC) [file pmed.1001253.s001.doc]

**Table S1. Ten largest food service companies in South Africa**

| Largest food service companies | Location of headquarters | Share of the market | Examples of chain brands owned by company |
| --- | --- | --- | --- |
| Famous Brands Ltd | South Africa | 20.0% | Wimpy, Debonairs Pizza |
| Yum Brands Inc | USA | 15.6% | KFC |
| Nando's Group Holdings | South Africa | 9.5% | Nandos |
| Spur Corp Ltd |  | 3.9% | Spur Steak Ranches, Panarottis Pizza |
| McDonald's Corp | USA | 2.8% | McDonalds |
| Golden Fried Chicken | South Africa | 2.5% | Golden Fried Chicken |
| King Pie Holdings | South Africa | 2.5% | King Pie |
| Taste Holdings | South Africa | 1.5% | Scooters, Maxis, St Elmos |
| Captain DoRego's Fast Foods & Fresh Fish | South Africa | 1.4% | Captain DoRego's' |
| Ocean Basket Group | South Africa | 1.3% | Ocean Basket |

Source: Euromonitor International, 2011 http://www.euromonitor.com/
