# Supplementary material for: “Big Food,” the Consumer Food Environment, Health, and the Policy Response in South Africa
Source: PLoS Med. 2012 Jul 3;9(7):e1001253. doi: 10.1371/journal.pmed.1001253 (PMC3389030; doi:10.1371/journal.pmed.1001253)
Supplement: Table S2 — Supermarket value sales, number of outlets, and company shares by value in South Africa 2007–2009. (DOC) [file pmed.1001253.s002.doc]

Table S2. Supermarket value sales, number of outlets, and company shares by value in South Africa 2007 -2009

|  | **2007** | **2008** | **2009** | **Supermarket brands owned by company** |
| --- | --- | --- | --- | --- |
| Number of supermarket outlets | 3 732 | 3 689 | 3 595 |  |
| Value sales from supermarkets, US$ million | 12 525 | 13 030 | 14 518 |  |
| % of retail value represented excluding sales tax |  |  |  |  |
| Shoprite Holdings | 34.8 | 39.4 | 41.6 | Shoprite Supermarkets, Checkers Supermarkets |
| Pick n Pay Retail Group | 38.8 | 36.7 | 34.6 | Pick n Pay Supermarkets, Pick n Pay Family Stores, Boxer, Score, Pick n Pay Minimarkets |
| Spar | 20.4 | 20.0 | 20.4 | Spar, Kwik Spar |
| Others | 5.9 | 3.9 | 3.5 |  |
| Total | 100 | 100 | 100 |  |

Source: Euromonitor In: USDA Foreign Agricultural Services (2011) GAIN Report South Africa 2010 Annual Retail Food Sector Report
